# Outbreak of poisoning by sodium hydrogen methylarsonate (MSMA)—an arsenic‐based herbicide—in horses in Brazil

**DOI:** 10.1111/evj.70132

**Published:** 2025-12-08

**Authors:** Gabriella Faria Pereira, Maria Clara Hornich Blimbliem, Anna Laura Previato Rosa Machado, Junara Bianca Rosa Abdala, Geison Morel Nogueira, Hugo Shisei Toma, Tatiane Furtado de Carvalho, Diego José Zanzarini Delfiol

**Affiliations:** ^1^ School of Veterinary Medicine and Animal Science Federal University of Uberlândia Uberlândia Brazil

**Keywords:** diarrhoea, horse, petechiae, toxicosis

## Abstract

**Background:**

Arsenic poisoning in horses is rarely reported in the literature. However, arsenic compounds can be present in rodenticides, pesticides, and herbicides, representing a potential source of accidental exposure for horses.

**Objective:**

To describe the epidemiological, clinical, and laboratory findings from a herd of 31 horses exposed to pasture treated with an overdose of sodium hydrogen methylarsonate, and to compare results in a group of horses exposed to the recommended dosage of the same product.

**Study Design:**

Observational field study.

**Methods:**

Thirty‐one horses were evaluated after grazing on a pasture sprayed with 7.9 kg/ha of MSMA. Sixteen animals underwent clinical examination, haematological and biochemical analyses, anatomopathological evaluation, and toxicological analysis. Arsenic concentrations were determined in biological and environmental samples to confirm exposure. The findings were compared with data collected from a group of horses grazing a paddock treated with the recommended dose.

**Results:**

The outbreak had a morbidity rate of 45.2% (14/31), a mortality rate of 19.4% (6/31), and a case fatality rate of 42.9% (6/14). Toxicological analysis showed increased arsenic concentrations in biological and environmental samples, confirming the diagnosis of poisoning. Supportive therapy was administered until the definite diagnosis and recovery of the surviving horses. Animals in the comparative group remained clinically normal and showed low arsenic concentrations.

**Main Limitations:**

The observational nature of the study, the delayed initiation of monitoring, and prior therapeutic interventions limited the scope of clinical data. Moreover, the rarity of arsenic poisoning in horses and the paucity of reference data restricted interpretation.

**Conclusions:**

Arsenic toxicosis in horses affects gastrointestinal, vascular, and renal systems. Diagnosis is supported by anatomopathological findings, biochemical alterations, and detection of arsenic in kidney, liver, and faecal samples.

## 1 INTRODUCTION

Sodium hydrogen methylarsonate (MSMA) is an arsenic‐based ingredient in organic contact herbicides. Arsenic is a metalloid present in rodenticides, insecticides, and herbicides. It can act as a toxic agent for domestic animals, especially those susceptible to food contaminated with arsenic compounds.^1^, ^2^, ^3^, ^4^ The pathogenesis of poisoning by arsenic compounds is related to their prompt absorption by the gastrointestinal tract of animals and rapid transport to tissues, where they reach the intracellular environment, react with sulphydryl molecular groups and inhibit the activity of glycolytic and mitochondrial enzymes involved in energy metabolism. This results in disruption of oxidative phosphorylation, and reduction of ATP production, critically affecting cellular respiration.^5^, ^6^, ^7^ Furthermore, arsenic compounds exert toxic effects through the generation of reactive oxygen species (ROS), associated with the inhibition of antioxidant enzymes. These ROS contribute to oxidative stress and cellular damage, particularly in organs with high metabolic and mitotic activity, such as the liver, kidneys, and intestinal epithelium.^2^, ^8^, ^9^

At the tissue level, these mechanisms result in widespread cellular death, resulting in the loss of capillary integrity, leading to haemorrhage and necrosis in various organs, and have a corrosive effect on the gastrointestinal mucosa, causing ulcers and local inflammation. Nephrotoxic effects have been reported, with signs of degeneration and renal tubular necrosis.^1^, ^6^, ^9^, ^10^, ^11^ The kidneys are highly affected not only because of their high oxidative activity, but also due to their ability to reduce absorbed pentavalent arsenic to the more toxic trivalent form, contributing to direct nephrotoxicity, which can be exacerbated by concomitant dehydration in profuse diarrhoea cases.^2^, ^9^, ^11^

Most cases of arsenic poisoning are reported in ruminants. Confirmed cases in horses are rare,^1^, ^12^ making the documentation of equine cases important for enhancing early diagnosis in this species. Therefore, the current report aims to describe the epidemiological, clinical, and laboratory findings of an outbreak of spontaneous MSMA poisoning in horses.

## 2 CASE REPORT

A herd of 31 horses was allocated to grazing paddocks where the MSMA‐based herbicide had been applied, 10 days prior to animal introduction. The herbicide was applied in two Tifton 85 paddocks, used for hay production. The area of Paddock 1 was 2.12 ha, and that of Paddock 2 was 1.86 ha. The herbicide was broadcast with a spraying system coupled to a tractor. Five litres of product diluted in 450 L of water were applied per ha of plantation per application. Two applications were made 7 days apart, resulting in a total administration of 7.9 kg of MSMA per ha. This amount is five times the dosage recommended on the product label.

All animals in the herd were inspected, and 16 were selected for detailed clinical and post‐mortem evaluation. The selection was primarily based on clinical relevance and logistics. After consuming the grass, 14 horses became intoxicated to varying degrees, beginning 10 days after they entered the paddocks. These 14 horses exhibited the clinical signs consistent with arsenic poisoning or presented post‐mortem findings compatible with intoxication. This group comprised several adults, as well as all foals in the herd and three pregnant mares. Two apparently healthy horses were included based on specific inclusion criteria: (1) the only asymptomatic pregnant mare (A16); and (2) a randomly selected, non‐pregnant adult horse (A15), included to serve as a reference.

**FIGURE 1:**
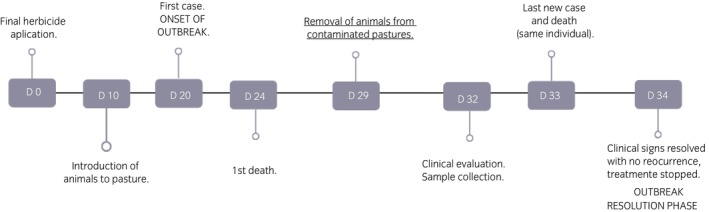
Timeline presenting the main events of sodium hydrogen methylarsonate (MSMA) poisoning in horses. D = day.

The correlation between the clinical signs and pasture exposure was only made on day 29 after herbicide application, when the animals were removed from the treated paddocks. A visit to the stud farm was carried out on day 32 and included a necropsy, clinical assessment of the surviving horses, and the collection of samples that confirmed the diagnosis of arsenic poisoning. The main events of the arsenic poisoning are presented in a timeline (Figure 1).

### 2.1 Clinical findings

Thirteen of the 31 exposed horses had clinical signs of arsenic poisoning, and another horse died without previously identifiable clinical signs (Table 1). The clinical signs varied in type and intensity and included profuse diarrhoea (13/14, 92.85%), colic (4/14, 28.57%), hypothermia (3/14, 21.42%), cyanosis (4/14, 28.57%), tongue protrusion (3/14, 21.42%), and difficulty in walking (3/14, 21.42%) characterised by ataxia progressing to recumbency and, ultimately, inability to rise. It was possible to categorise the cases into distinct clinical presentations based on the duration of the clinical course, the severity of the clinical signs, and the rapidity of disease progression (Table 1). The presentations observed were acute, subacute, and asymptomatic.

**TABLE 1:** Signalment, clinical signs identified, outcome, clinical course duration and clinical presentation classification of 16 horses exposed to pasture in which a high dosage of sodium hydrogen methylarsonate had been applied.

Animal	Sex	Age	Breed	Clinical signs/post‐mortem alterations	Outcome	Clinical course duration	Clinical presentation
A1	M	16Y	QH	Diarrhoea, colic	Survival	13 days	Subacute
A2	F	10Y	QH	Diarrhoea	Survival	10 days	Subacute
A3	F	2Y	QH	Diarrhoea	Survival	10 days	Subacute
A4	M	11M	PONY	No clinical signs/ Multisystemic inflammation, intestinal and pulmonary haemorrhage, and hepatic alterations with bile duct proliferation.	Death	<24 h	Acute
A5	F	13Y	PONY	Diarrhoea	Death	<24 h	Acute
A6	F	5M	QH	Diarrhoea	Survival	7 days	Subacute
A7	F	4M	QH	Diarrhoea	Survival	8 days	Subacute
A8	F	5M	QH	Diarrhoea	Survival	6 days	Subacute
A9	F	12Y	QH	Diarrhoea, colic, hypothermia, cyanosis, tongue protrusion, impaired mobility	Death	36 h	Acute
A10	F	21Y	MM	Diarrhoea, colic, hypothermia, cyanosis, tongue protrusion, impaired mobility/Widespread haemorrhage, nephrosis with tubular necrosis, steatosis, and multisystemic inflammation.	Death	5 days	Acute
A11	F	17Y	QH	Diarrhoea	Survival	6 days	Subacute
A12	F	15Y	MM	Diarrhoea colic, hypothermia, cyanosis, tongue protrusion, impaired mobility	Death	24 h	Acute
A13	F	15Y	MM	Diarrhoea	Survival	6 days	Subacute
A14	F	12Y	MM	Diarrhoea, cyanosis	Death	<24 h	Acute
A15	F	3Y	QH	No clinical signs	Survival	—	Asymptomatic
A16	F	14Y	QH	No clinical signs	Survival	—	Asymptomatic

Abbreviations: F, female; M, male; M, months; MM, Mangalarga Marchador; QH, Quarter Horse; Y, years.

The acute presentation included animals that either experienced sudden death or rapid disease progression over up to 5 days. Some horses had a rapid clinical course, with death occurring in less than 24 h after the observation of initial mild or nonspecific signs. This was the case for horse A4, an 11‐month‐old pony that died without prior clinical signs, and horse A5, a 13‐year‐old pony with diarrhoea and death within a few hours. Similarly, horse A14 was last observed with no clinical signs but was found dead 14 h later; liquid faeces were noted on its haircoat and the mucous membranes were diffusely cyanotic. Other animals in this category, however, had longer clinical courses with a broader range of clinical signs. These signs initially included diarrhoea and abdominal pain, progressing to hypothermia, cyanotic mucous membranes, tongue protrusion, and ambulatory difficulty before death. This progressive course was observed in animals A9, A10, and A12 (a pregnant mare).

The subacute presentation was characterised as a more prolonged clinical course (6–13 days), with primarily gastrointestinal signs and lower mortality. The horses in this category showed profuse diarrhoea as their main clinical sign, with only one horse (A1) additionally showing colic, and subacute cases survived. Intestinal hypermotility was identified by auscultation of the four abdominal quadrants, and petechiae in the ocular and nasal mucosa were observed in these individuals. This group consisted of three suckling foals (A6, A7, and A8) and several adults, including two pregnant mares (A2 and A11).

Animals that were exposed to the contaminated pastures but did not present clinical signs were classified as asymptomatic and consisted of one pregnant mare (A16) and one non‐pregnant mare (A15). The pregnant mares' clinical, gestational period at the time of outbreak, pregnancy outcome, and survival status are described in Table 2.

**TABLE 2:** Gestational phase at the time of outbreak, clinical signs presented, and gestational and clinical outcome of four pregnant mares exposed to pasture. in which a high dosage of sodium hydrogen methylarsonate had been applied.

Animal	Gestational period	Clinical signs	Gestational and clinical outcome
A2	7 months	Diarrhoea	Full‐term pregnancy resulting in healthy foal. Survival of the mare.
A11	5 months	Diarrhoea	Abortion at 7 months of gestation. Survival of the mare.
A12	5 months	Diarrhoea, colic, hypothermia, cyanosis, tongue protrusion, impaired mobility	Death of the foetus and mare.
A16	7 months	No clinical signs	Full‐term pregnancy resulting in healthy foal. Survival of the mare.

### 2.2 Ancillary diagnostic tests

#### 2.2.1 Clinical pathology

Complete blood counts (CBCs) were performed on six horses (A1, A2, A10, A11, A13, and A15) revealing only minor alterations in most cases (Table S1). Serum biochemistry was assessed in seven animals (A1, A2, A10, A11, A12, A13, and A15). All animals had increased creatinine concentrations (ranging from 178 to 701 μmol/L), and urea increased in all but one horse (A10). Four animals (A1, A2, A8, and A10) showed hypoalbuminemia, and one (A9) had gamma‐glutamyl transferase (GGT) activity above the reference range (39.8 U/L). Hyperbilirubinemia was found in A1 (total: 51.5; indirect: 48.1 μmol/L), an animal that was clinically stable at the time of evaluation but previously presented diarrhoea and colic. Creatine kinase (CK) values were slightly increased in A2 and A12, and lactate concentrations were notably increased in A12 (23 mg/dL). Complete serum biochemistry results are available in Table S2. Venous blood gas samples from four animals (A1, A2, A11, and A13) were evaluated: hyponatremia was observed in three of the four horses, hyperglycaemia and decreased bicarbonate concentrations in one horse (A10), and increased bicarbonate concentrations in another horse (A1). Complete venous blood gas results are available in Table S3.

Urinalysis was performed on a sample from horse A2, collected via bladder catheterization. The alterations observed were slightly increased specific gravity (1.051), acidic pH (5.5), and the presence of occult blood (1+), urobilinogen (1+), bilirubin (1+), and clusters of transitional cells (1+).

#### 2.2.2 Pathological examinations

**FIGURE 2:**
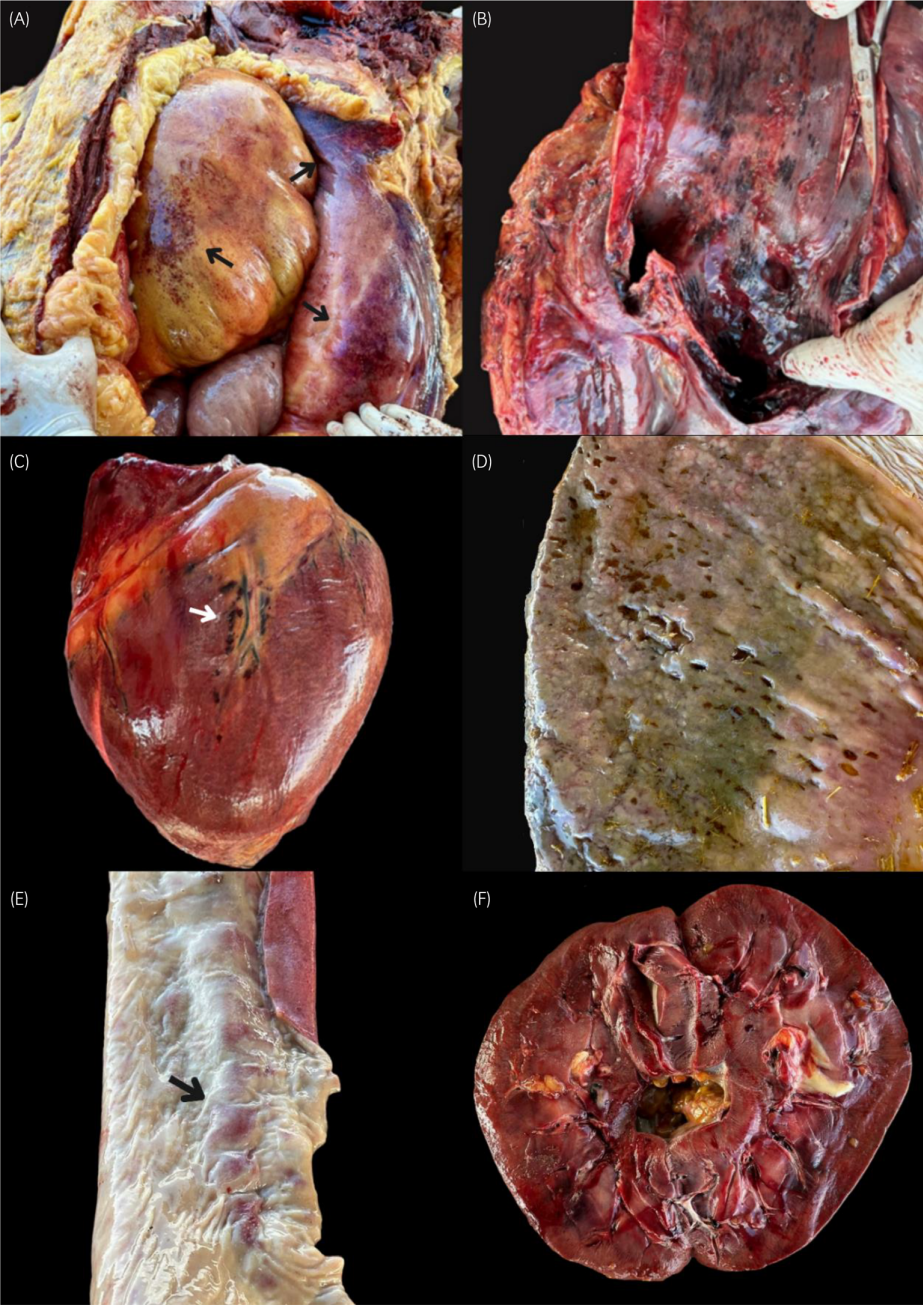
Main macroscopic alterations observed in horse A10 poisoned with sodium hydrogen methylarsonate, including petechiae and ecchymoses in the large intestine (A), trachea (B), and epicardium (C). Intense gastric erosion (D), and red areas in the intestinal mucosa (E) and renal parenchyma (F).

Horse A10 underwent post‐mortem examination on the property 8 h after death. Upon opening the abdominal cavity, a large amount of fibrinous reddish fluid was present. Areas of petechiae and ecchymosis were observed in the peritoneum, small and large intestine (Figure 2A), mesentery, trachea (Figure 2B), lungs, and epicardium (Figure 2C). The gastric mucosa showed ulcerations (Figure 2D) present in a large part of the glandular portion of the organ, and the remaining portion of the epithelium was hyperemic. The gastric contents were liquid, cloudy in appearance, and orange‐red in colour. The large intestine had a thickened mucosa, with multifocal haemorrhagic areas (Figure 2E), and mucus was present. When cut, the kidney showed multifocal dark red spots, measuring 0.5 cm in diameter, distributed in the cortical and medullary regions (Figure 2F). The liver was diffusely pale and enlarged, with discreetly yellowish and friable multifocal areas.

#### 2.2.3 Histopathological examination

Peritoneum, lung, kidney, liver, adrenal gland, heart, stomach, spleen, brain, urinary bladder, ovary, uterus, duodenum, and colon samples were collected from horse A10. Samples of small intestine, large intestine, lungs, liver, and stomach from horse A5 were available for histopathology. The tissues were stained with haematoxylin and eosin (HE).

**FIGURE 3:**
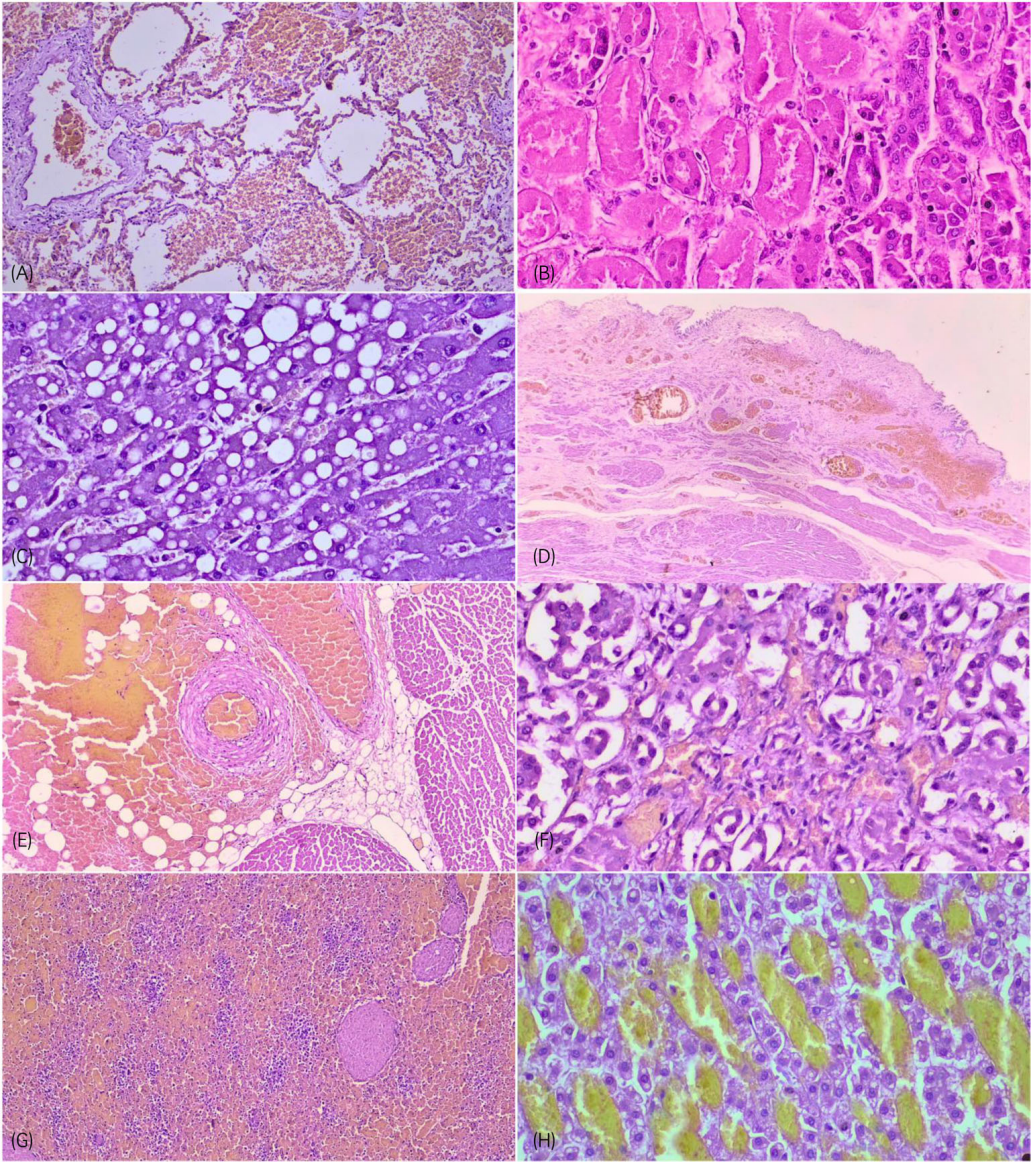
Haematoxylin and eosin‐stained organ tissue from horse (A10) poisoned by sodium hydrogen methylarsonate. (A) Prominent haemorrhage in the lungs, 10× magnification. (B) Nephrosis and renal tubular necrosis, with numerous tubules showing swollen cells, pyknosis and karyolysis, 40× magnification. (C) Intracytoplasmic vacuolisation in hepatocytes moderately and diffusely moving the nucleus to the periphery, characterising steatosis, 40× magnification. (D) Urinary bladder mucosa with marked haemorrhaging, 4× magnification. (E) Marked haemorrhaging in the epicardium, 10× magnification. (F) Mild congestion and haemorrhaging in the kidneys, 4× magnification. (G) Moderate congestion in the spleen, 10× magnification. (H) Adrenal gland, 40× magnification.

Histopathological examination of organ samples from horses A10 and A5 revealed multisystemic alterations. In horse A10, extensive areas with marked haemorrhaging were observed in the peritoneum, associated with a discrete multifocal lymphocytic inflammatory infiltrate and intense hyperaemia. In the lungs, there was a discrete multifocal lymphocytic inflammatory infiltrate in the interstitium, alveolar spaces were filled with amorphous eosinophilic fluid, and marked multifocal to coalescent haemorrhages were present (Figure 3A). Similarly, in horse A5, there was moderate haemorrhaging in the lungs.

In the kidneys of horse A10, the proximal tubules had large cytoplasm, increased eosinophilia, and small, poorly delimited intracytoplasmic vacuoles. Pyknosis and karyolysis were sometimes evident. There was detachment of cells into the tubular lumen, compatible with nephrosis and tubular necrosis (Figure 3B). In the renal interstitium, there was a mild multifocal lymphohistiocytic inflammatory infiltrate.

In the gastrointestinal tract, both horses showed inflammatory changes. Horse A10 exhibited a moderate diffuse lymphocytic inflammatory infiltrate in the mucosa of the duodenum and colon, with moderate multifocal haemorrhages in the colon fragments. In horse A5, a moderate and diffuse inflammatory infiltrate composed of lymphocytes, plasma cells, histiocytes, and eosinophils was observed in the mucosa of the large intestine. There were multifocal and coalescent areas of deposition of amorphous eosinophilic material, with cellular debris in the submucosa, associated with a pronounced and diffuse inflammatory infiltrate composed of lymphocytes, rare plasma cells, histiocytes, and eosinophils. In the small intestine and stomach, there was a moderate to intense lymphocytic inflammatory infiltrate in the interstitium. Severe haemorrhaging was also observed in horse A5's large intestine.

In the liver of horse A10, there was intracytoplasmic vacuolisation in hepatocytes, moderately and diffusely moving the nucleus to the periphery (Figure 3C). In horse A5's liver, a moderate inflammatory infiltrate composed of lymphocytes was observed predominantly in the periportal region, associated with a discrete and diffuse proliferation of bile ducts. The hepatocytes presented a discrete increase in volume and diffusely granular cytoplasm.

The mucosa of the urinary bladder in horse A10 exhibited marked erosions, haemorrhages (Figure 3D), and oedema, with discrete multifocal neutrophilic inflammatory infiltrate. In this horse, there was marked haemorrhaging in the epicardium (Figure 3E), with discrete haemorrhages in the ovary and kidney (Figure 3F). Moderate to severe congestion was observed in the brain, liver, lungs, kidneys, spleen (Figure 3G), adrenal gland (Figure 3H), stomach, urinary bladder, colon, and uterus. Horse A10's spleen also showed marked diffuse lymphoid depletion.

#### 2.2.4 Toxicological analysis

Arsenic concentrations were measured in blood samples from four symptomatic horses (A1, A2, A11, and A13), and one asymptomatic horse (A15), the urine from one symptomatic horse (A2), the faeces from two symptomatic horses (A2 and A13), and the kidney and liver samples from a symptomatic, necropsied horse (A10). Soil, grass and water samples were collected from the two paddocks in which the horses were exposed to high dosages of MSMA. The samples were obtained through a composite sampling process. Five separate areas within each paddock were selected, and representative portions of each area were collected, followed by unification into a single sample (per paddock). This process yielded one composite soil sample and one composite grass sample per paddock. A water sample was collected from a masonry water trough with an automatic float, which was accessed by animals in both high‐dosage paddocks. All samples were subjected to arsenic quantification using absorption spectrometry.

Results of arsenic concentration in the biological samples are available in Table 3. The results were above acceptable concentrations in kidney, liver, urine and faecal samples. The soil samples had arsenic concentrations of 136.8 ppm (Paddock 1) and 45.5 ppm (Paddock 2). The pasture samples had arsenic concentrations of 369.3 ppm (Paddock 1) and 147.9 ppm (Paddock 2). The water sample contained <0.5 ppm of arsenic.

**TABLE 3:** Arsenic concentration in biological samples from horses exposed to pasture treated with an overdosage of sodium hydrogen methylarsonate (POISONING OUTBREAK) and horses exposed to label‐compliant dosages of sodium hydrogen methylarsonate (comparative group).

Sample type	Source	Sample	Arsenic concentration (ppm)	Reference values
Tissue fragment	Poisoning outbreak	Kidneys A10	29.1	<10 ppm^24^
		Liver A10	31.9	
Whole blood	Poisoning outbreak	A1	<0.05	≤0.015 ppm^a^
		A2	<0.05^a^	
		A11	<0.05	
		A13	<0.05	
		A15	<0.05	
	Comparative group	CG1	<0.05	
		CG2	<0.05	
Faeces	Poisoning outbreak	A2	14.7	^b^
		A13	22.5	
	Comparative group	CG1	0.15	
		CG2	0.06	
Urine	Poisoning outbreak	A2	0.409	≤0.3 ppm^a^
	Comparative group	CG1	<0.05	
		CG2	<0.05	

Abbreviations: A, animal; CG, comparative group; ppm, parts per million.

^a^ Values refer to thresholds established for anti‐doping purposes and are not intended for clinical diagnosis of arsenic poisoning. However, due to the lack of toxicological reference values in the literature, they are presented here as comparative references.

^b^ Reference values not found in the literature.

#### 2.2.5 Treatment

Treatment was initiated at the onset of clinical signs, despite the absence of a definitive diagnosis, as animals began to experience diarrhoea or abdominal pain. Analgesia was administered using flunixin meglumine (1.1 mg/kg, intravenous), particularly in horses with abdominal discomfort or signs compatible with systemic inflammation. Fluid therapy with Ringer's lactate solution was used in several cases, with infusion rates adjusted according to clinical severity (based on intensity and frequency of diarrhoea, or signs of shock).

As the outbreak progressed, strategies based on the clinical suspicion of a non‐specific toxic aetiology were applied, including the administration of activated charcoal (80 mg/kg, once daily), oral probiotics (12 mg/kg, once daily), intravenous fluid supplemented with B‐complex vitamins (2 mL/kg, once daily), and a liver‐supporting compound containing essential amino acids and metabolic cofactors (0.1 mL/kg of Mercepton, intravenous, once daily). The frequency and duration of these supportive and protective therapies varied according to clinical response but were often maintained for at least 3 days. The decision to implement this therapy was based on the onset or recurrence of diarrhoea. The antimicrobial combination of sulfadoxine and trimethoprim (15 mg/kg, intramuscular, once daily) was included in the protocol mainly when the risk of secondary bacterial infections was suspected.

Outcomes from treatment varied among individuals. Some horses, such as those treated early with gastrointestinal adsorbents and liver protectants, showed progressive improvement. Other horses, particularly those with rapid neurological decline or prolonged hypoperfusion, had their clinical condition deteriorate despite intervention. Treatment in all horses was discontinued by day 34, with complete resolution of clinical signs without recurrence. The animal owners provided follow‐up information 3 months after resolution of the outbreak, reporting no health complications observed in the evaluated animals, except for abortion in horse A11 2 months after the poisoning episode.

## 3 HORSES EXPOSED TO CONVENTIONAL DOSES OF THE HERBICIDE

As MSMA‐based herbicides are commonly used in the control of weeds in Tifton pastures in the region of the outbreak, environmental samples and samples from horses grazing a paddock sprayed with the recommended dosage of the product, were collected aiming to better understand the outbreak presented in this report. A 2.0‐ha Tifton 85 paddock was sprayed with 2 L/ha of diluted MSMA‐based herbicide, resulting in a total administration of 1.58 kg of MSMA per ha (label‐compliant dosage). A herd of 10 horses was reintroduced into the pasture 10 days after application and remained there until sampling day.

Samples were collected 58 days after herbicide application, and 48 days after animal reintroduction, and submitted for arsenic concentration analysis. Soil and grass samples were collected through a composite sampling process as described in Section 2.2.4. Biological samples were collected from two randomly selected animals and no animal developed clinical signs after exposure to the pasture. The selected animals were CG1, a 16‐year‐old Quarter Horse mare and CG2, a 12‐year‐old Quarter Horse mare. Blood, urine and faeces were collected, being submitted for CBC, serum biochemistry and arsenic concentration analysis.

Physical examination was unremarkable in the two selected animals and CBC and serum biochemistry were unremarkable. Results from arsenic quantification in environmental samples were 0.07 0.07 ppm in the soil sample and <0.05 ppm in the grass samples. Arsenic analysis is shown in Table 3.

## 4 DISCUSSION

We describe an outbreak of MSMA poisoning in horses due to the consumption of grass sprayed with an arsenic‐based herbicide. The horses showed diarrhoea and renal dysfunction, evidenced by increased serum creatinine concentrations, as the main clinical alterations. The key factors affecting the degree of poisoning included the quantity and frequency of herbicide application, the time of exposure to the agent, and the individual characteristics of the animals that may have contributed to their susceptibility to the toxic agent.

MSMA is recommended for use in cotton and sugarcane crops, with the aim of controlling various weeds. The product label advises the use of a single application at a dosage of 1.8–3 L of diluted product per ha. In our report, 10 L (7.9 kg) of MSMA were applied per ha, over a 7‐day period. This corresponds to a dosage 3–5 times greater than the label recommendation, constituting off‐label usage and leading to significant environmental contamination.^4^, ^13^

Although arsenic poisoning in horses is described in toxicology manuals, published case reports remain scarce, with few descriptive articles in the literature.^1^, ^6^, ^9^, ^11^, ^14^ The arsenic poisoning reported here, with acute, subacute, and asymptomatic presentations, points to a key factor for the development of clinical signs in horses after arsenic consumption: the dosage. The role of dosage in the pathogenesis of arsenic poisoning is further reinforced by the contrast between horses on the overdose pastures and the comparative group horses. In the comparative group paddock, a fifth of the product (1.58 kg/ha) was applied to the pastures, resulting in no affected animals. Although it was not possible to determine the amount of grass ingested by each individual, the spectrum of clinical presentations observed in this report is consistent with published descriptions of the clinical signs of arsenic toxicosis as dosage‐dependent, with high amounts producing neurological signs and sudden death, and lower dosages producing gastrointestinal signs and renal dysfunction, with the possibility of a more prolonged condition, and even asymptomatic animals.^5^, ^15^

The technical guidelines for the product, originally intended for label‐specified crops, recommend a preharvest interval of at least 43 days (for cotton), with an indefinite interval for sugarcane, due to its multiple final destinations. A restricted‐entry interval of at least 7 days is also recommended.^4^ In our study, the horses were placed in paddocks 10 days after the last MSMA application, resulting in intoxication in the horses exposed to an increased dosage of MSMA, and no clinical alterations in the comparative group horses. In this context, the absence of specific guidelines for the reintroduction of grazing animals hinders interpretation of the waiting period's adequacy. Nonetheless, the evidence suggests that the amount of product applied to the pasture, rather than reintroduction timing, dictated the clinical outcome.

Susceptibility to arsenic poisoning is also linked to other factors, such as exposure time, species, age, and individual characteristics, such as genetic factors, concomitant diseases, and nutritional status. Studies indicate that younger animals, as well as those that are weaker, debilitated, or dehydrated, are more susceptible to arsenic poisoning.^11^, ^16^ In our study's herd exposed to an overdose of MSMA, 42% of the horses were symptomatic, but 100% of the overexposed horses under 1 year of age were affected, with one of them not surviving.

Interestingly, among the overexposed animals, only two were ponies and both died acutely: one without previously observed clinical signs and the other <24 h after the onset of diarrhoea. This mortality might be attributed to a greater intake of the intoxicating agent proportional to their live weight, since nutritional studies show that ponies have a higher consumption of forage per kg of live weight than other types of horses.^17^

Regarding the toxic effects of arsenic on the reproductive system, there are reports of an increase in cases of abortion in sows and cows following episodes of environmental contamination by arsenic, as well as experimental trials with mice indicating that arsenic leads to spontaneous abortion in mammals by inducing changes in placental vasculogenesis.^11^, ^18^ In our study, one of the overexposed pregnant mares aborted 2 months after feeding on the overdosed pasture. At the time of the outbreak, the mare that aborted was 5 months pregnant, while the mares with surviving offspring were at 7 months of gestation. Placental development and vascularisation are still incomplete at 5 months, with key angiogenic maturation processes still occurring,^19^ whereas by 7 months these processes are more advanced. This physiological difference may render the placenta more vulnerable to the disruptive vascular effects of arsenic at earlier stages, potentially leading to structural and functional compromise that is incompatible with long‐term foetal survival.

The CBCs in these animals had few noteworthy changes and are not a test of choice in arsenic poisoning diagnosis. Horse A10's erythrocytosis and leukopenia were interpreted as haemoconcentration secondary to dehydration and attributed to gastrointestinal inflammation and this horse had confirmed enteritis.^11^ Additional alterations such as anaemia (horse A11) and thrombocytosis (horses A11 and A10) were observed, but are unspecific. Given the lack of a baseline haematological evaluation, it is not possible to definitively attribute these changes to arsenic exposure alone.^20^, ^21^

Serum biochemistry indicated renal injury, with increased creatinine and urea concentrations in all overexposed horses, even those that were asymptomatic. Arsenic causes renal damage through its direct action on the kidney, and through hypoperfusion resulting from dehydration and shock generated by its action on other organs.^22^, ^23^ Additionally, arsenic promotes the disruption of oxidative phosphorylation, the kidneys' role in filtering blood, generation of ROS, and reducing pentavalent arsenic to its more toxic trivalent form.^2^, ^6^, ^7^, ^9^

Hyperbilirubinaemia in horse A1 occurred during the recovery phase at the time of sampling may reflect residual effects of prior hepatic dysfunction, whereas the increase in gamma‐glutamyl transferase observed in horse A9 prior to its fatal outcome can be interpreted as an active hepatic lesion. Hepatic damage is a known consequence of arsenic toxicosis, as the compound disrupts cellular energy production and promotes oxidative overload, leading to hepatocellular stress. This process may manifest as biochemical alterations or histological changes, such as the fatty degeneration of hepatocytes (steatosis) observed in horse A10.^7^, ^9^, ^24^

The hypoalbuminaemia found in four could relate to several alterations resulting from arsenic's activity, such as enteric, renal and hepatic damage.^2^, ^24^, ^25^ Creatine kinase activity was mildly increased but not considered clinically relevant.^26^

Anatomopathological examination is highly useful for the diagnosis of arsenic toxicosis, as it reveals histological lesions that strongly support the diagnosis in affected animals. The most prominent changes are seen in the gastrointestinal tract, including haemorrhages due to capillary degeneration, as well as areas of necrosis in the gastrointestinal mucosa and submucosa, and fluid gastrointestinal contents. Furthermore, renal tubular necrosis and fatty degeneration of hepatocytes are generally consequences of the nephrotoxicity and hepatotoxicity of arsenic, which are compatible with the changes we observed in the two horses subjected to post‐mortem analyses.^6^, ^9^, ^24^

To assess toxicological samples, the regional screening concentrations developed by the US Environmental Protection Agency (2024) can be used as a reference. Regional screening concentrations provide cut‐off values for the concentration of environmental contaminants, acting as guidelines that indicate the need for further investigation or actions. The regional screening concentrations for inorganic arsenic in residential and industrial soils are 6.8 and 3 ppm, respectively. The concentrations in our soil samples from both overdosed paddocks (136.8 and 45.5 ppm) are significantly higher than the established screening values.^27^ These increased values contrast with the arsenic concentration found in the comparative group paddock. In this case, the sample was collected 58 days after herbicide application, 26 days later than the sampling in the overdosed paddocks, which occurred 32 days post‐application. The arsenic concentration in the comparative group paddock sample was 0.07 ppm, significantly lower than the values in the overdosed paddocks. This analysis is particularly valuable, as it reflects the baseline arsenic concentrations expected in the region's soil following a label‐compliant application of MSMA. It provides a reference point that incorporates both the natural background concentration and any residual impact from a recent, regulated exposure, thereby reinforcing the abnormality and significance of the increased arsenic concentrations found in the overexposed sites.

The European Union directive 2002/32/EC (2002) can be used as a reference range for the amount of arsenic in animal pasture. It establishes that forage intended for animal consumption must not contain more than 2 ppm of arsenic compounds, aiming to avoid accumulation in products of animal origin, but also to avoid illnesses associated with chronic arsenic intake.^28^ An outbreak of acute poisoning that led to the death of 14 Girolando cattle due to MSMA toxicosis resulted in arsenic concentrations of 111.58 ppm in the grass analysed.^29^ The arsenic concentrations in the pasture samples analysed in this study were 147.9 and 369.3 ppm, higher than those reported in these two references. The same comparison between soil arsenic concentrations applies to grass arsenic concentrations, with concentrations in the overdosed paddocks far surpassing those detected in the comparative group paddock, where arsenic was below the detection limit (<0.05 ppm) of the analytical method used.

In tissue samples, a definite diagnosis of arsenic poisoning can be made when concentrations greater than 10 ppm are detected in liver and kidney fragments.^22^ The liver and kidney samples had concentrations of 31.9 and 29.1 ppm, respectively, which serve as a sensitive marker for the diagnosis of toxicosis caused by arsenic compounds. In contrast, whole blood samples, collected 72 h after animal removal from the overexposed pastures showed arsenic concentrations below the analytical threshold (<0.05 ppm). The only horse‐specific comparative reference found for plasma and urine in the literature relates to anti‐doping control in sport horses, where the cut‐off point for total arsenic in plasma is established at 0.015 ppm.^30^ However, this value is intended to detect unauthorized use of arsenic‐based tonics and not for diagnosis of clinical toxicosis, making direct comparison limited. Therefore, while the anti‐doping threshold is used here as a comparative value, a scientifically defined diagnostic cut‐off for arsenic poisoning in horses has yet to be established.

Urine samples collected 72 h after horse removal from the overexposed pastures showed arsenic concentrations slightly above the anti‐doping threshold (0.409 ppm; cut‐off: 0.3 ppm). While such thresholds are not designed for clinical diagnosis, but rather to detect doping‐level arsenic concentrations up to 22 h after intravenous administration, the persistence of increased concentrations beyond the expected elimination window suggests substantial exposure.^30^ Our findings also corroborate literature data that arsenic compounds are rapidly eliminated from the blood and urine of animals, with half‐lives of 38 and 44 h in urine and plasma, respectively. Consequently, these biological samples are not ideal for diagnosis but can be used when exposure to arsenic has not been interrupted for over 38 h.^31^

Arsenic concentrations in faeces also appeared to be significant in both horses evaluated. Unfortunately, there are no data available regarding expected values in non‐intoxicated horses. Arsenic concentrations continued to be detectable, although in much lower concentrations, in faeces from the comparative group horses. The contrast between the arsenic concentrations in the faecal, urine, and blood samples points to faecal samples as a better option for toxic compound quantification in cases where exposure to the agent has been interrupted for more than 38 h.^31^, ^32^

When comparing the diagnostic value of evaluated biological samples, tissue analysis was the most definitive, especially for retrospective confirmation in fatal cases, with arsenic concentrations clearly exceeding established toxicological thresholds. Faecal analysis showed moderate diagnostic sensitivity and potential practical value, particularly when tissue collection is not feasible, although the lack of established reference ranges limits its interpretation. In contrast, blood and urine were less helpful due to the time elapsed between exposure and sampling, despite a high likelihood they could be shown to present in the acute‐phase.^30^ These differences highlight the importance of selecting diagnostic tests based on the timing of sample collection and the stage of exposure. The combined interpretation of environmental and biological samples significantly improved the overall confidence of the diagnosis in this outbreak.

Regarding the treatment of MSMA poisoning, the first measure is to eliminate the exposure to the source of toxicity. However, this action depends on an established diagnosis and recognised contaminant source, which in most cases occurs later than ideal.^6^ In this outbreak the connection between the clinical signs and the pasture was made belatedly, resulting in horses continuing to graze the overdosed pastures for 19 days. Concerning the effectiveness of the therapeutic interventions instituted, which were mainly supportive and nonspecific, it was possible that the combination of fluid therapy, hepatic support and oral gastrointestinal adsorbent administration may have increased survival chances in subacute cases. However, horses with shorter acute presentations did not allow for intervention in a timely manner resulting in negative outcomes even with intensive therapy. Our findings reinforce the assertion that early recognition of clinical signs and the toxic agent is essential for better therapeutic results and a lower number of non‐survivors.^11^, ^12^

The specific therapy for arsenic poisoning is sodium thiosulfate, aiming to reduce the toxicity of the arsenic, to protect sulfhydryl groups (the primary targets of arsenic molecules) and exert antioxidant action. Orally administered sodium thiosulfate binds to arsenic in the gastrointestinal tract, preventing arsenic's absorption, an action potentially beneficial in the evaluated animals as traces of arsenic were still present in the faeces. When administered intravenously, the drug increases arsenic excretion, mainly via urine, thereby reducing its toxic action on cellular enzymes and possibly resulting in slower progression of clinical signs.^5^, ^33^ Due to commercial unavailability neither the owner nor the veterinary team was able to acquire sodium thiosulfate in the adequate pharmaceutical formulation and quantities for equine treatment.^5^, ^33^ In the absence of a specific therapy, supportive treatment includes monitoring of renal and gastrointestinal function, avoiding further exposure of the horses to herbicide‐treated fields, and correcting potential electrolyte imbalances as needed.^6^, ^15^, ^34^

Our case report has several limitations inherent to the nature and timing of our involvement in the poisoning by MSMA. The arsenic intoxication event had been ongoing for 32 days before our team was involved, at which point most animals were already in the resolution phase. Consequently, we were unable to monitor the clinical progression from the onset of signs or to request comprehensive diagnostic testing across all affected animals. Additionally, as therapeutic interventions had already been initiated prior to our arrival, our role was limited to retrospective documentation of the treatment protocols employed. The rarity of arsenic poisoning in horses further constrained our interpretation, given the scarcity of comparable reports and the lack of well‐established reference values for arsenic concentrations in equine biological samples.

We conclude that arsenic poisoning in horses can present with different intensities, with the main clinical signs related to the gastrointestinal system. The most relevant laboratory alterations are related to renal function, whereas in anatomopathological examinations, vascular, renal, and gastrointestinal mucosal lesions are highlighted. Furthermore, in toxicological evaluation, kidney and liver samples are useful for diagnosis, and faecal analysis shows potential diagnostic value.

## FUNDING INFORMATION

Support for publication was received from the Coordenação de Aperfeiçoamento de Pessoal de Nível Superior ‐ Brasil (CAPES) (ROR identifier: 00x0ma614).

## CONFLICT OF INTEREST STATEMENT

The authors have declared no conflicting interests.

## AUTHOR CONTRIBUTIONS

**Gabriella Faria Pereira:** Data curation; investigation; formal analysis; visualization; writing – original draft. **Maria Clara Hornich Blimbliem:** Investigation; visualization. **Anna Laura Previato Rosa Machado:** Investigation; resources. **Junara Bianca Rosa Abdala:** Investigation; resources. **Geison Morel Nogueira:** Investigation; writing – review and editing. **Hugo Shisei Toma:** Writing – review and editing. **Tatiane Furtado de Carvalho:** Investigation; writing – review and editing. **Diego José Zanzarini Delfiol:** Conceptualization; investigation; writing – review and editing; supervision.

## DATA INTEGRITY STATEMENT

Gabriella Faria Pereira had full access to all the data in the study and takes responsibility for the integrity of the data and the accuracy of the data analysis.

## ETHICAL ANIMAL RESEARCH

This report was approved by the Ethics Committee on Animal Use (CEUA) under protocol number 23117.051388/2023‐08 for hospital care of animals.

## INFORMED CONSENT

Informed consent was obtained from the animals’ owner for the publication of this report.

## Supporting information


**Table S1.** Complete blood count of six horses exposed to pasture applied with a high dosage sodium hydrogen methylarsonate.

**Table S2.** Serum biochemistry analysis of blood from animals exposed to pasture contaminated with an overdosage of sodium hydrogen methylarsonate (MSMA).

**Table S3.** Haemogasometry results from venous blood samples from animal exposed to pasture contaminated with an overdosage of sodium hydrogen methylarsonate (MSMA).

## Data Availability

The data that support the findings of this study are available upon reasonable request from the corresponding author. Open data sharing exemption granted by the editor.
