# Supplementary material for: Outbreak of poisoning by sodium hydrogen methylarsonate (MSMA)—an arsenic‐based herbicide—in horses in Brazil
Source: Equine Vet J. 2025 Dec 8;58(5):1258–68. doi: 10.1111/evj.70132 (PMC13447871; doi:10.1111/evj.70132)
Supplement: Supplementary file 1 [file EVJ-58-1258-s001.pdf]

**Table S3:** Haemogasometry results from venous blood samples from animal exposed to pasture contaminated with an overdosage of sodium hydrogen methylarsonate (MSMA).

| ANIMAL/ PARAMETER | $Na^+$ (mmol/L) | $K^+$ (mmol/L) | $Cl^-$ (mmol/L) | iCa (mmol/L) | GLU (mg/dL) | pH T | $HCO_3^-$ (mmol/L) |
|---|---|---|---|---|---|---|---|
| **A1** | 135.4 | 3.98 | 103.4 | 1.45 | 77 | 7.43 | 34.6 |
| **A2** | 130.9 | 4.78 | 106.6 | 1.46 | 73 | 7.37 | 25.2 |
| **A8** | 125.8 | 4.86 | 100.2 | 1.40 | 78 | 7.40 | 28.2 |
| **A10** | 128.7 | 3.83 | 106.4 | 1.43 | 161 | 7.35 | 20.9 |

Na+ - sodium; K+ - potassium; Cl- - Chloride; iCa – ionised calcium; pH T – pH corrected for temperature; $HCO_3^-$ - bicarbonate.
