# Supplementary material for: Outbreak of poisoning by sodium hydrogen methylarsonate (MSMA)—an arsenic‐based herbicide—in horses in Brazil
Source: Equine Vet J. 2025 Dec 8;58(5):1258–68. doi: 10.1111/evj.70132 (PMC13447871; doi:10.1111/evj.70132)
Supplement: Supplementary file 2 [file EVJ-58-1258-s002.pdf]

Equine Veterinary Journal BEVA

Table S1: Complete blood count of six horses exposed to pasture applied with a high dosage sodium hydrogen methylarsonate.

ANIMAL/ PARAMETER	RBC ($x10^6$/uL)	Hb (g/dL)	PCV (%)	WBC	NEU	EOS	BAS	MON	LYM	PLT ($x10^3$/uL)
A1	7.48	12.6	35	7,400	5,550	74	0	148	1628	214
A2	7.66	12.6	37	7,000	3,360	700	0	280	2660	234
A7	14.24	22.7	70.4	4,300	5,322	0	0	172	1806	165
A8	5.37	9.8	30	5,400	4,158	162	0	162	918	699
A10	8.87	15.4	46	7,800	5,850	156	0	312	1482	479
A12	7.82	12.1	35	7,000	3,500	350	140	70	2940	124
REFERENCE VALUES	6.0 – 11.3	10.6 – 18.9	34 - 49	5.29 – 13.75 x 10^3	2,700 – 9,650	60 - 580	10 - 160	130 - 590	1,100 – 5,690	100 - 350

RBC – red blood cells; Hb – haemoglobin; PCV – packed cell volume; WBC – white blood cell count; NEU – neutrophil count; EOS – eosinophil count; BAS – basophil count; MON – monocyte count; LYM – lymphocyte count; PLT –platelet count. All cell counts refer to the number of cells in 1 µL of sample. Reference values provided by the Veterinary Clinical Laboratory (LCVET), Veterinary Hospital, Federal University of Uberlândia (UFU), Uberlândia, Brazil.
