# Supplementary material for: Outbreak of poisoning by sodium hydrogen methylarsonate (MSMA)—an arsenic‐based herbicide—in horses in Brazil
Source: Equine Vet J. 2025 Dec 8;58(5):1258–68. doi: 10.1111/evj.70132 (PMC13447871; doi:10.1111/evj.70132)
Supplement: Supplementary file 3 [file EVJ-58-1258-s003.pdf]

Equine Veterinary Journal BEVA

Table S2: Serum biochemistry analysis of blood from animals exposed to pasture contaminated with an overdosage of sodium hydrogen methylarsonate (MSMA).

ANIMAL/ PARAMETER	ALB (g/dL)	AST (U/L)	GGT (U/L)	CREA (mg/dL)	UREA (mg/dL)	TBIL (mg/dL)	DBIL (mg/dL)	IBIL (mg/dL)	CK (U/L)	LAC (mg/dL)
**A1**	2.68	220	7.2	2.33	48	3.01	0.20	2.81	356	9
**A2**	3.31	234	7.3	2.87	39.5	0.65	0.30	0.35	444	6
**A7**	-	318.5	15	7.93	-	-	-	-	-	-
**A8**	2.17	248	6.2	2.27	73.6	0.81	0.35	0.46	270	13
**A9**	-	322.1	39.8	3.13	-	-	-	-	-	-
**A10**	2.55	211	7.4	2.22	26	1.19	0.41	0.78	365	16
**A12**	2.78	302	6.9	2.01	41	0.84	0.32	0.52	403	23

ALB – albumin; AST – aspartate aminotransferase; GGT – gamma-glutamyl transferase; CREA – creatinine; TBIL – total bilirubin; DBIL – direct bilirubin; IBIL – indirect bilirubin; CK – creatine kinase; LAC – lactate.
